# Event‐ and Time‐Based Prospective Memory and Time Perception in Autistic Adults With and Without Intellectual Disabilities

**DOI:** 10.1002/aur.70250

**Published:** 2026-04-09

**Authors:** Daniela Nürnberg, Mareike Altgassen

**Affiliations:** ^1^ Department of Psychology Johannes Gutenberg University Mainz Mainz Germany

**Keywords:** high‐functioning autism, intellectual disability, low‐functioning autism, prospective memory, time perception

## Abstract

The aim of this study was to examine time perception (i.e., the sense of the duration, order and passage of time) and event‐ and time‐based prospective memory (PM; i.e., the ability to recall an intention to perform an action in the future) in adults across the wider autism spectrum including those with intellectual disabilities. Sixty‐four adults, aged 18–65 years, took part in the study (32 autistic, 32 neurotypical controls). Participants were tested using computerized event‐ and time‐based PM tasks and two different time perception tasks (naturalistic vs. comprehensive consisting of time estimation, time production and time reproduction). Results showed no differences between the groups in both PM tasks after controlling for verbal abilities. Moreover, both groups displayed comparable performance in event‐ versus time‐based PM tasks. Autistic participants showed difficulties in time perception in comparison to neurotypical controls in the naturalistic, but not in the comprehensive time perception task. In both groups better performance in time perception was linked to better event‐ and time‐based PM performance. Given the high relevance of PM in everyday life, further research is needed to support autistic individuals in their day‐to‐day lives. This is one of the first studies investigating PM in autistic adults, including those with intellectual disabilities, a part of the spectrum that is often overlooked.

## Summary

Time perception, how someone understands the passing of time, and prospective memory, the ability to remember what you want to do in the future, were investigated in autistic and non‐autistic participants with and without intellectual disabilities. The prospective memory performance was the same for both groups. Autistic participants had difficulties with naturalistic time perception compared to non‐autistic controls. For both groups, performance in prospective memory and time perception was related; better memory for future intentions was associated with better time perception. A better understanding of time might help autistic individuals improve their prospective memory.

## 1 Introduction

Autism spectrum disorder (ASD) is a neurodevelopmental disorder characterized by impaired social communication and interaction, coupled with the presence of restricted, repetitive interests, activities, or behaviors (American Psychiatric Association 2013). Furthermore, co‐occurring conditions, such as intellectual disabilities (ID) or limited expressive language, can increase the severity of impairments (Waizbard‐Bartov et al. 2023). Based on these co‐occurring conditions and the degree to which autistic individuals experience difficulties with cognitive, verbal, motor, social, and adaptive skills, they can be categorized as rather “low”‐ or “high‐functioning.” Individuals with low‐functioning autism (LFA) usually show ID and limited language skills; characteristics that are mostly not observed in individuals with high‐functioning autism (HFA; Kamp‐Becker et al. 2020). Moreover, individuals with LFA exhibit greater severity of ASD core characteristics, less adaptive skills, and require often extensive lifelong support compared to individuals with HFA (Cohen et al. 2014).

Autistic individuals often show difficulties with memory tasks, such as short‐term memory and free recall (Desaunay et al. 2020), as well as item memory (Ring et al. 2016). Furthermore, prospective memory (PM) difficulties have been documented in autistic individuals (Landsiedel et al. 2017; Sheppard et al. 2018). PM refers to the cognitive processes involved in planning specific actions in the future, as well as in the self‐initiated recall and execution of these actions (Brandimonte et al. 1996; Kliegel and Jäger 2006). PM comprises multiple processes and phases (Kliegel et al. 2002). The individual forms an intention for the future and stores it in retrospective memory while concurrently performing other ongoing tasks. At the appropriate moment, these ongoing activities must be inhibited, and the individual needs to switch to the prospective action and execute it as planned (Ellis 1996; Sheppard et al. 2018). PM tasks can be distinguished based on the cue indicating the appropriate moment for task initiation. For event‐based PM tasks, an intended action needs to be remembered when a specific target stimulus is presented (e.g., remembering to buy milk when seeing the supermarket). For time‐based PM tasks, the action has to be performed at a designated point in time or after a specified period of time has elapsed (e.g., remembering an appointment at 3:00 pm; Kliegel and Jäger 2006).

There is evidence of reduced event‐ and time‐based PM in individuals with HFA compared to typically developing (TD) controls in laboratory studies. For example, Altgassen et al. (2012) compared autistic adults to age‐ and ability‐matched controls in the “Dresden Breakfast Task,” in which participants had to prepare breakfast with real objects following certain rules and time restrictions and completing embedded event‐ and time‐based PM tasks. Results showed that controls outperformed autistic participants in event‐ and time‐based PM tasks. Consistently, Kretschmer et al. (2014) compared autistic adults with controls in the “Virtual Week,” a computerized game that simulates everyday tasks. Controls performed better than autistic adults on event‐ and time‐based PM tasks. Similarly, a subsequent Virtual Week investigation demonstrated reduced performance of autistic adults in event‐ and time‐based PM compared to TD controls; importantly, the difficulties in time‐based PM were larger than in event‐based PM (Dehnavi and Khan 2024).

Other studies have identified differences between autistic and control participants in time‐based but not in event‐based PM tasks. Time‐based PM tasks put greater demands on executive functions as there is typically no external cue that may prompt retrieval of the intended action, and the participant is required to actively keep in mind the elapsing time (Brandimonte et al. 1996). For instance, Williams et al. (2014) tested autistic and non‐autistic adults in a computerized PM task. For the ongoing task, participants should memorize words and make yes‐or‐no recognition judgments. For the time‐based task, participants were required to press a specific button at 2‐min intervals. For the event‐based task, they had to press a specific button whenever the word presented was a musical instrument. The performance of autistic adults was reduced on time‐based but not on event‐based PM compared to neurotypical controls. Consistently, using the same computerized task, Landsiedel and Williams (2020) found that autistic adults demonstrated lower performance in time‐based PM compared to TD controls. A further study by Charlton et al. (2023) observed more self‐reported PM difficulties with the “Prospective and Retrospective Memory Questionnaire” in autistic compared to non‐autistic adults.

Taken together, most studies have observed PM difficulties in adults with HFA compared to age‐ and ability‐matched TD controls. Recent studies by Faustmann and Altgassen (2024, 2025) and Groenman et al. (2024) found spared PM performance. This may be due to the broadening of diagnostic criteria for autism that led to an increased number of individuals being diagnosed with ASD (Hull et al. 2020; Zeidan et al. 2022) and to more subgroups of autistic individuals (Mottron and Bzdok 2020). This development presents a potential opportunity for individuals with milder autistic characteristics—as compared to earlier diagnosed individuals—to participate in research studies which may mitigate potential group differences. However, all these previous studies included adults with HFA and no autistic participants with ID. To date, only one study by Sheppard et al. (2016) also tested children with LFA. Their findings indicated lower performance of children with LFA, but not with HFA, in event‐based PM tasks compared to TD children. Further research is needed to explore whether adults with LFA encounter similar challenges in PM.

The reported greater difficulties in time‐based compared to event‐based PM in ASD have often been related to the higher executive function demands of time‐based PM (Einstein and McDaniel 1996). However, individuals' ability to perceive time may also represent a crucial component of time‐based PM (Glicksohn and Myslobodsky 2006). Time perception is defined as the ability to comprehend the duration of an event or stimulus, the temporal order of stimuli, and the passage of time (Casassus et al. 2019). Common time perception tasks are time estimation, time production and time reproduction. For time estimation tasks, participants are typically asked to estimate the number of seconds of a given time interval. For time production tasks, the exact number of seconds of a time interval is given with participants tasked with generating this interval. The act of imitating a specific time interval, e.g., directly from the experimenter, constitutes the process of time reproduction (Wallace and Happé 2008). Several studies report reduced time perception in autism (Jurek et al. 2019). For example, Brenner et al. (2015) compared children and adolescents with HFA and TD controls and found lower time reproduction in autistic individuals. Brodeur et al. (2014) examined the time estimation of brief durations (less than 1 s) in children with LFA and TD children. Participants with ASD exhibited reduced sensitivity to variability in short durations compared to TD children. Similarly, Falter et al. (2012) investigated adolescents and adults with HFA compared to TD controls in a temporal generalization task and found a reduced sensitivity for temporal discrimination in individuals with ASD compared to TD controls. In a parent reported study, parents of autistic and neurotypical children completed the “It's About Time” questionnaire which assesses the child's sense of time (e.g., “How does your child prepare so they can do things, such as going to school, on time?”). Overall, results indicated reduced time‐related behaviors in autistic children as compared to TD controls. Importantly, autistic children experienced difficulties with *prospective cognition* (i.e., difficulties in contemplating the future and preparing for forthcoming events; Poole et al. 2021). In contrast, another study by Poole et al. (2022) found spared timing performance of autistic adults compared to non‐autistic controls on a battery of auditory and visual timing tasks. Although self‐reported “It's About Time” questionnaire scores indicated greater difficulties in time perception of autistic adults, deficits were not observed in their timing performance.

Despite the extensive research conducted on the relationship between time perception and (retrospective) memory (Block and Grondin 2014; Matthews and Meck 2016) and their neural overlap (Üstün et al. 2017), there are only a few studies investigating the relationship between time perception and memory in autistic individuals. Time perception has been demonstrated to influence the performance of autistic individuals in episodic memory tasks (Maister and Plaisted‐Grant 2011). As PM comprises both temporal elements and memory processes, an association with time perception could be assumed. To date, only a few studies assessed the role of time perception on PM, and none of those included autistic participants. Mioni et al. (2017) examined children with ADHD and controls and found that time perception was a significant predictor of PM accuracy. Consistently, Mioni et al. (2012) compared adults suffering from traumatic brain injury and matched controls and found that in the control group time reproduction performance was associated with time‐based PM accuracy. Similarly, Mackinlay et al. (2009) investigated neurotypical school‐aged children in time‐based PM and time estimation and production and found a significant correlation between time perception and time‐based PM accuracy. In contrast, Mioni and Stablum's (2014) study with younger and older adults revealed that time perception was a significant predictor of time‐monitoring behavior during a time‐based PM task and not a predictor of PM accuracy. To date, no study has investigated the relationship between time perception and PM in ASD.

Even though one‐third of autistic individuals exhibit an ID (Zeidan et al. 2022) and approximately 23% demonstrate borderline ID (Jack and Pelphrey 2017), since about the 1980s research has primarily focused on autistic individuals without ID (Chakrabarti 2017; Jack and Pelphrey 2017; Lombardo et al. 2019). The dearth of research on autistic individuals with ID, as well as their dependence on external assistance in daily living, underscores a crucial need to explore this research domain further. Therefore, we aimed to investigate time perception and event‐ and time‐based PM performance in autistic and non‐autistic adults with and without ID. In addition, we aimed to investigate the relationship between time perception and PM, as successful PM execution requires both temporal monitoring and memory processes. By examining this link in autistic adults, the current study contributes to understanding how differences in temporal cognition might influence PM performance in this population. In line with previous research (Landsiedel et al. 2017), we expected autistic adults to show lower PM performance compared to TD controls. We expected both groups to perform better on event‐ than time‐based PM, with autistic participants showing greater difficulties on time‐based PM compared to TD controls (Williams et al. 2014). Moreover, we expected autistic individuals to show reduced time perception compared to TD controls (Brenner et al. 2015; Brodeur et al. 2014; Falter et al. 2012). We expected time‐based PM performance to be related to participants' perception of time (Mioni et al. 2017).

## 2 Methods

### 2.1 Participants

A total of 70 adults were tested, comprising 36 individuals with an ASD diagnosis and 34 neurotypical controls. ASD participants were recruited in residential groups and sheltered workshops for people with mental disabilities via mailing lists and flyers, and control participants additionally via neighborhood apps. In a first step, autistic participants were recruited. Thereafter, control participants were recruited trying to match them closely to autistic participants in terms of age, gender, and education level. The inclusion criteria were as follows: participants were required to be aged between 18 and 65 years, to be native German speakers, and for those in the ASD group, to have received a clinical diagnosis of ASD (date of diagnosis was not collected). Exclusion criteria comprised the presence of severe psychiatric or neurological disorders, the intake of psychotropic substances and a lack of speech. Six participants were excluded from the study due to severe cognitive or physical impairment which made task completion impossible. Finally, 32 adults with an ASD diagnosis (26 male, 5 female, 1 diverse) and 32 control participants without an ASD diagnosis (27 male, 5 female) were included into data analysis. In the ASD group, 34.4% indicated a comorbid mental disorder (most frequent affective disorders, ADHD and PTSD/anxiety disorders), 40.6% had no comorbid mental disorder, while 25% refused to respond. In the control group, 18.7% reported a comorbid disorder, predominantly affective disorders. The control group was parallel to the autism group in terms of chronological age, gender and non‐verbal abilities on the matrices reasoning subtests of the Wechsler Adult Intelligence Scale‐Fourth Edition (WAIS‐IV). A significant discrepancy was identified in verbal abilities, with controls demonstrating superior verbal skills in comparison to the autistic group (see Table 1).

**TABLE 1:** Participants characteristics.

	ASD (*n* = 32) *M* (SD); range	Controls (*n* = 32) *M* (SD); range	*F* (df)	*η* ^2^
Age	35.9 (12.2); 19–65	38.2 (13.3); 18–64	0.527 (1,62)	0.008
NVA age‐scaled scores	6.8 (5.4); 1–19	7.3 (3.1); 1–13	0.231 (1,62)	0.004
VA age‐scaled scores	5.3 (4.5); 1–13	8.0 (3.4); 1–14	7.423 (1,62)**	0.107
SCQ total score	*n* = 24^a^ 25.4 (5.0); 17–35	/	/	/

Abbreviations: NVA, non‐verbal ability; VA, verbal ability.

** *p* < 0.01.

^a^ The SCQ was not completed by three parents. In addition, five caregivers were unable to complete the section of the questionnaire pertaining to the participant's childhood because they did not know the participant during their childhood, and the parents were either deceased or unreachable. Consequently, the total score for these eight participants is missing.

Participants were compensated for their participation in the study (10 EUR per hour). Prior to their participation, all participants and parents or caregivers of participants with external guardianship provided written informed consent. The study was conducted in accordance with the Declaration of Helsinki and approved by the local university ethics committee.

### 2.2 Materials

#### 2.2.1 Autism Severity

The FSK (Fragebogen zur sozialen Kommunikation, sozialen Interaktion und stereotypen Verhaltensweisen; Bölte et al. 2006) is a German version of the Social Communication Questionnaire (SCQ; Rutter et al. 2003) comprising 40 items. It is used as an external assessment to evaluate autism severity completed by parents or long‐term carers. The internal consistency of the overall scale is *α* = 0.83, and the retest reliability (6 months to 2 years) is rtt = 0.76. The response procedure is dichotomous (“yes” or “no”). The first 19 items assess the participant's current behavior, while the final 21 items refer to the participant's behavior at the age of 4–5 years. The questions are divided into three categories: social communication, social interaction and stereotypical behavior. The maximum score is 39 points; the standard cut‐off score for an autism diagnosis is 16 points.

#### 2.2.2 Verbal Abilities

To evaluate the verbal abilities of the participants, the vocabulary subtest of the German version of the WAIS‐IV was administered (Wechsler 2008). The vocabulary subtest assesses an individual's lexical knowledge and their capacity to verbally articulate the meanings of words. As the task progresses, the level of difficulty increases with each word. Raw scores were converted to age‐scaled scores.

#### 2.2.3 Non‐Verbal Abilities

To evaluate the non‐verbal abilities of the participants, the matrices reasoning subtest of the German version of the WAIS‐IV was administered (Wechsler 2008). The matrices reasoning subtest requires participants to identify patterns within a given design. With the progression of the task, the difficulty increases with each matrix. Raw scores were converted to age‐scaled scores.

#### 2.2.4 Prospective Memory

**FIGURE 1:**
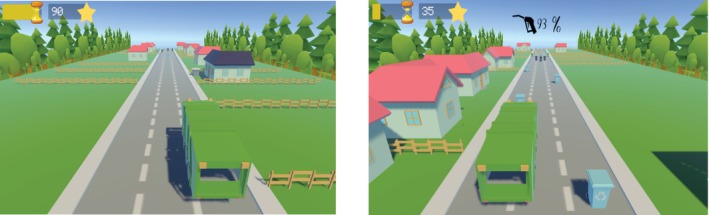
Mainz garbage truck task. *Note*: The event‐based cue (blue roof) can be seen on the left, while the refueling indicator (93%) is shown on the right as a time‐based cue.

PM performance was assessed with a computerized task called the “Mainz Garbage Truck Task.” For the ongoing activity, participants were required to navigate a garbage truck using the arrow keys on a street collecting light blue garbage cans, while avoiding dark blue and green garbage cans and obstacles. To assess time‐based PM, participants were requested to remember to fill up the gas whenever the fuel level dropped below 10% by pressing the “B”‐key. The fuel level gradually diminished from 100% to zero across a time span of 45 s. Participants could check the fuel level by pressing the space bar (measuring time monitoring) visible for 3 s. To measure event‐based PM, participants were instructed to collect special garbage cans in front of houses with blue roofs whenever they passed one by pressing the “M”‐key. All other houses had red roofs (see Figure 1). The event‐based PM task was designed as a non‐focal paradigm (McDaniels and Einstein 2000) putting higher demands on strategic monitoring. This allowed the task to impose similar demands on executive functions as the time‐based task. Prior to the experimental round, participants were required to complete a practice round of about one and a half minutes to ensure task comprehension. The total time of the experimental block took about 6 min. There were five time‐based and five event‐based PM tasks embedded (in total 10 PM tasks). Dependent variables were PM performance (ranging from 0 to 10 points), the number of fuel checks, and performance on the ongoing task, calculated as the difference between the number of garbage cans collected correctly and incorrectly. The task was conducted at the lowest possible speed (70% of the normal speed).

#### 2.2.5 Time Perception

For a *naturalistic* perception of time, participants were required to verbally indicate the number of minutes that had elapsed since the start of the test session at three time points during the test procedure (after having completed specific tasks; approximately after 15, 40, and 60 min). The time deviation from the correct time was measured in seconds.

The assessment of time estimation, time production, and time reproduction (termed *comprehensive* time perception in this study) was conducted based on the study by Wallace and Happé (2008). For the time estimation task, the experimenter specified time intervals (4, 12, and 45 s), commencing with the word “Go” and concluding with the word “Stop”. Participants were tasked with estimating the number of seconds that had elapsed between these verbal commands. For the time production task, participants were asked to produce specific time intervals (4, 12, and 45 s). Participants were required to say “Go” and after, for example, 4 s, they had to say “Stop.” Finally, for the time reproduction task, participants were asked to imitate time intervals (4, 12, and 45 s) that were presented by the experimenter. The sequence of the three time perception types was randomized among participants. A stopwatch was used to measure the time intervals and answers of the participants. The time deviation from the correct time was measured in seconds.

### 2.3 Procedure

Testing took place in the laboratory of the local university or at the participant's residence, contingent upon the participant's preference. Compared to controls, autistic participants demonstrated a clear preference for home testing (19 autistic participants and four controls were tested at home, while 13 autistic participants and 28 controls were tested in the laboratory). The test room was a calm space without clocks. Participants were requested to remove their wristwatches and to place their mobile phones out of sight. Testing was conducted one‐on‐one, unless the participant requested the presence of a parent or caregiver. All tests were conducted by the same experimenter to increase comparability. Firstly, the consent form was signed by each participant, the experimenter and, in instances of external guardianship, also by the parents or caregivers. Then the vocabulary subtest was administered. This was followed by the comprehensive assessment of time perception. After this, participants completed the Mainz Garbage Truck Task and finally, the matrices subtest. The three measurement points for the naturalistic time perception were conducted at predetermined time points. The SCQ was completed by parents (*n* = 24) or by long‐term carers (*n* = 5) before or during the laboratory test. Importantly, carers only completed the section regarding current behavior given they had not known the participants during childhood.

## 3 Results

### 3.1 PM Performance

To analyze the effects of type of PM task (event‐based vs. time‐based) and group (ASD vs. Controls) on PM performance, a 2 × 2 mixed analysis of variance (ANOVA) was conducted. There was no significant interaction effect between group and PM task, *F* < 1. The main effect of group was significant, *F* (1,62) = 6.38, *p* = 0.014, *η*_p_^2^ = 0.093. There was no significant main effect of type of PM task, *F* < 1, indicating no differences in performance for event‐ and time‐based PM tasks of both groups. As verbal abilities differed between groups, a separate analysis of covariance (ANCOVA) was conducted to control for verbal abilities (for a similar procedure, see Altgassen et al. 2008). The significant group effect disappeared, *F* (1,61) = 1.16, *p* = 0.286, *η*_p_^2^ = 0.019, indicating no group differences between autistic and non‐autistic participants in PM when controlling for verbal abilities. Univariate ANOVAs were conducted to investigate fuel monitoring during the PM task and ongoing task performance. Controls monitored the fuel level more often than autistic individuals, *F* (1,62) = 4.75, *p* = 0.03, *η*_p_^2^ = 0.07. After controlling for verbal abilities with a separate ANCOVA, the significant group effect disappeared, *F* (1,61) = 1.03, *p* = 0.315, *η*_p_^2^ = 0.017. There was no difference between the groups regarding the ongoing task performance, *F* (1,62) = 1.35, *p* = 0.25, *η*_p_^2^ = 0.02.

### 3.2 Time Perception

#### 3.2.1 Naturalistic Task

To analyze the effects of the three different measurement time points (1 vs. 2 vs. 3) and group (ASD vs. Controls) on estimation accuracy, a 3 × 2 mixed ANOVA was conducted. There was a significant interaction effect between group and time points, *F* (1.21,67.25) = 4.46, *p* = 0.034, *η*_p_^2^ = 0.069. The main effect of measurement time points was significant, *F* (1.21,67.25) = 13.16, *p* < 0.001, *η*_p_^2^ = 0.180, as well as the main effect of group, *F* (1,60) = 9.30, *p* = 0.003, *η*_p_^2^ = 0.134. After controlling for verbal abilities with a separate ANCOVA, the significant interaction effect disappeared, *F* (1.13,66.87) = 1.89, *p* = 0.173, *η*_p_^2^ = 0.031, but the significant main effects of measurement time points, *F* (1.13,66.87) = 15.55, *p* < 0.001, *η*_p_^2^ = 0.209, and group, *F* (1,59) = 4.996, *p* = 0.029, *η*_p_^2^ = 0.078, remained. Pairwise comparisons indicated that both groups exhibited deteriorations with each measurement time point (*p* = < 0.001, *p* = 0.018, *p* < 0.001), with the autistic group demonstrating lower performance compared to controls.

#### 3.2.2 Comprehensive Task

To analyze the effects of type of time perception (time estimation vs. time production vs. time reproduction), duration of time in seconds (4 vs. 12 vs. 45), and group (ASD vs. Controls) on accuracy, a 3x3x2 mixed ANOVA was conducted. There were no significant interaction effects between group and type of task, *F* < 1, between group and duration, *F* (1.07,64.45) = 1.47, *p* = 0.235, *η*_p_^2^ = 0.024, between type and duration, *F* (1.39,83.43) = 2.81, *p* = 0.084, *η*_p_^2^ = 0.045, and between group, type and duration, *F* < 1. The main effect of type of time perception was significant, *F* (1.19,71.16) = 7.58, *p* = 0.005, *η*_p_^2^ = 0.112, as well as the main effect of duration, *F* (1.07,64.45) = 53.19, *p* < 0.001, *η*_p_^2^ = 0.470. Both groups showed superior performance in time reproduction in comparison to time production (*p* = 0.006) and time estimation (*p* = 0.008) with no difference between time production and time estimation (*p* = 0.10). Pairwise comparisons demonstrated that each measurement time point differed significantly from the others (*p* < 0.001), with the most accurate results observed at 12 s and the poorest at 45 s for both groups. There was no significant main effect of group, *F* < 1, (see Table 2).

**TABLE 2:** Mean and standard deviations of performance in PM and time perception.

	ASD (*n* = 32) *M* (SD)	Controls (*n* = 32) *M* (SD)
PM performance		
Event‐based PM	1.97 (2.02)	2.87 (1.66)
Time‐based PM	1.69 (1.86)	2.78 (1.81)
PM fuel monitoring	6.63 (6.37)	10.50 (7.79)
PM ongoing task	110.84 (62.54)	127.16 (48.26)
Time naturalistic	851.83 (909.55)	346.82 (218.49)
Time comprehensive		
Time estimation	9.90 (12.32)	9.74 (14.31)
Time production	7.34 (4.93)	6.10 (4.61)
Time reproduction	6.58 (5.83)	3.36 (3.56)

Abbreviation: PM, prospective memory.

### 3.3 PM and Time Perception

Pearson correlational analyses were carried out to explore the relation of PM performance with time perception (see Tables 3 and 4). In both groups, better time reproduction was associated with better time‐based PM. Furthermore, better time production and time reproduction were associated with better event‐based PM. There was no association between naturalistic time perception or time estimation and PM performance.

**TABLE 3:** Pearson correlations for autistic participants.

	Time‐based PM	Event‐based PM	Time naturalistic	Time estimation	Time production
Event‐based PM	0.512**				
Time naturalistic	−0.344	−0.201			
Time estimation	−0.266	−0.116	0.093		
Time production	−0.333	−0.509**	0.258	0.547**	
Time reproduction	−0.606***	−0.614***	0.360	0.630***	0.708***

Abbreviation: PM, prospective memory.

** *p* < 0.01.

*** *p* < 0.001.

**TABLE 4:** Pearson correlations neurotypical controls.

	Time‐based PM	Event‐based PM	Time naturalistic	Time estimation	Time production
Event‐based PM	0.442*				
Time naturalistic	−0.249	−0.007			
Time estimation	−0.146	−0.157	0.433*		
Time production	−0.164	−0.359*	0.198	0.618***	
Time reproduction	−0.399*	−0.559***	0.273	0.158	0.480**

*Note*: *p < 0.05; **p < 0.01; ***p < 0.001.

Abbreviation: PM, prospective memory.

## 4 Discussion

This study aimed to investigate time perception and PM performance in autistic adults with and without ID compared to age‐ and ability‐matched neurotypical controls.

### 4.1 PM Performance

Contrary to our assumption, we observed no differences in PM performance of neurotypical controls and autistic adults after controlling for verbal abilities. These findings contradict the vast literature examining adults with HFA (Altgassen et al. 2012; Kretschmer et al. 2014), and the only study investigating event‐based PM in LFA children (Sheppard et al. 2016), but they are in line with more recent studies that also did not observe group differences (Faustmann and Altgassen 2024, 2025; Groenman et al. 2024). Importantly, a group difference was observed in the mean values, with autistic participants demonstrating lower performance in both PM tasks compared to the control group. However, due to the large variance in the data and the disparity in verbal abilities among the groups, this difference was not statistically significant. The group difference in fuel monitoring also disappeared after controlling for verbal abilities. Future studies of the wider autistic spectrum should concentrate on larger samples and ensure equivalent verbal abilities among participants.

Moreover, in contrast to our hypothesis all participants performed comparably on the event‐ and time‐based PM tasks. There was also no significant interaction between type of PM task and group. The extant literature on this aspect provides mixed results. Regarding neurotypical individuals, there is evidence that participants perform better in event‐based compared to time‐based PM tasks (Khan et al. 2008; McBride and Flaherty 2020). However, conflicting results have also been reported (Wójcik et al. 2022). With respect to autistic adults, some studies found comparable performance in event‐ and time‐based PM tasks (Altgassen et al. 2012; Kretschmer et al. 2014), while others observed spared event‐based but reduced time‐based PM performance (Williams et al. 2014). An explanation for the absence of any differences between the two task types may be attributable to the characteristics of the Mainz Garbage Truck Task. Here, the presentation of event‐ and time‐based PM cues was simultaneous to the ongoing task. Consequently, it is plausible that performances on event‐ and time‐based tasks influenced each other. For instance, if participants detected a blue roof (event‐based task) and remembered to execute the intended action, this may have reminded them to monitor the fuel level and refuel the garbage truck (time‐based task). Consequently, the two measures may not be independent of each other and therefore be comparable in terms of performance, as is evident from the strong positive correlation between them (*r* = 0.515; *p* < 0.001). Future studies should investigate the nature of PM performance in participants with and without ID when event‐ and time‐based PM tasks are surveyed independently.

### 4.2 Time Perception

We expected time perception to be reduced in autistic adults compared to neurotypical controls. This hypothesis was partially confirmed. Autistic adults demonstrated lower performance in the *naturalistic* time perception task with a significant decrease in accuracy at each measurement point. This finding is consistent with earlier research in autistic adults (Falter et al. 2012; Jurek et al. 2019). In contrast, we found no differences between groups in the *comprehensive* time perception task based on the study by Wallace and Happé (2008), which is in contrast with previous research on time reproduction (Brenner et al. 2015) and time estimation (Brodeur et al. 2014), where difficulties were found in autistic children and adolescents in comparison to control groups. However, in line with our findings, Wallace and Happé (2008) did not report reduced time perception in children and adolescents with HFA. The contradicting results across studies may be due to the use of different measures of time perception. In the two studies in which a discrepancy between the groups was identified, computerized tasks were utilized (Brenner et al. 2015; Brodeur et al. 2014), in contrast, the study by Wallace and Happé (2008) employed face‐to‐face testing with an experimenter, as this study did. Another explanation could be the age of the participants. Brenner et al. (2015) found that younger children with ASD exhibited greater difficulties in time reproduction than older ones. In the study by Brodeur et al. (2014), where group differences were found, only younger children were investigated (mean age 10 years for ASD and 6 years for the control group). In contrast, in studies where no group differences were found, adolescents (Wallace and Happé 2008) and adults with ASD (Poole et al. 2022) were tested. Therefore, it is possible that difficulties in time perception decrease with increasing age in individuals with ASD, contributing to the missing group effect in our study. Moreover, the type of time perception task may have influenced the outcomes. Our analysis revealed group differences in the naturalistic but not in the comprehensive time perception task. This discrepancy could be attributed to the distinct demands placed by each task, despite both measuring time perception. The naturalistic task is more closely aligned with everyday life, in which individuals tend to be less conscious of time, as in the testing procedure they did not know when and how often they were asked to indicate the minutes elapsed so far. Moreover, the estimation of minutes and hours is closer to everyday life than the estimation of seconds. Consequently, in future studies, it may be beneficial to adopt time tasks that closely resemble everyday life, thereby facilitating a more authentic and ecologically relevant experimental environment for research.

### 4.3 PM and Time Perception

As expected, increased time‐based PM performance was associated with increased performance in time perception. In both groups, better performance in time reproduction was related to better time‐based PM performance. This result is consistent with the previous findings in participants with ADHD and neurotypical controls (Mioni et al. 2012, 2017). Surprisingly, in the present study, we found no correlation between time estimation as well as time production and time‐based PM, in contrast to earlier results in neurotypical children (Mackinlay et al. 2009). The differing results may be attributable to methodological discrepancies, including the time‐based PM tasks utilized, the time‐perception tasks, and the time intervals taken into consideration. For instance, the study by Mackinlay et al. (2009) employed a one‐back picture task as an ongoing task, wherein participants were required to press a yellow key every 2 min to measure time‐based PM. All other studies made use of a cartoon movie as an ongoing task, with participants required to press a key every 5 min. In addition, Mackinlay et al. (2009) utilized a set of four different tasks to measure time production and time estimation, whereas all other studies used only one time reproduction task. The heterogeneity of tasks and procedures may have impacted the outcomes, and comparability is limited. Moreover, the available findings do not include data on autistic participants, thereby highlighting a crucial need for further research in this domain.

Furthermore, in the present study, better event‐based PM was associated with better time production and time reproduction, which was not expected. As mentioned, this may be because the event‐ and time‐based PM tasks within the Mainz Garbage Truck Task are probably not independent. The observed association between time perception and event‐based PM highlights the need to include event‐based paradigms in future research on temporal cognition. Since real‐world tasks often blend external cues with internal time monitoring (e.g., baking a cake without a timer), investigating this interplay could be essential for an ecologically valid understanding of prospective memory in autism. If further research clarifies the connections between PM and time perception, results of such studies could offer a framework for practical applications, e.g., targeted training in time reproduction or in time estimation in everyday life to strengthen PM.

### 4.4 Limitations and Future Research

A difference in verbal abilities was observed between the groups. Consequently, the impact of verbal abilities was considered in the calculations where group differences were identified (PM, naturalistic time perception, time monitoring). Future studies should pay close attention to ensuring equal verbal abilities between groups, as these abilities can have a significant impact on PM performance, as demonstrated in our study. Moreover, it should be noted that the time production and reproduction methods inherently involve motor noise (verbal responses), which may lead to larger uncertainty in timing measurements compared to pure estimation tasks (Shi et al. 2013). However, since the testing conditions were identical for both groups, this factor probably influenced all participants to a similar extent and is therefore unlikely to have systematically biased the group comparison. Furthermore, only a single measurement was recorded for each time duration in the naturalistic time perception task and for each time type and time duration in the comprehensive time perception task which may have reduced reliability. This approach was adopted to minimize testing duration and number of testing sessions, given the sensitivity of the sample, which included individuals with ID. Future studies should consider measuring several trials per time type and time duration potentially by utilizing multi‐session testing to maintain feasibility. Additionally, to minimize total testing time, we did not use further questionnaires to verify the reported co‐occurring conditions. Future studies could control co‐occurring conditions, especially ADHD which may negatively impact PM performance. In general, there is an absence of tasks that are specifically designed for individuals with ID. In our own investigation, we have encountered favorable experiences with tasks that can adapt their level of difficulty to the individual being evaluated (see matrices or vocabulary subtest) and naturalistic tasks with higher ecological validity (e.g., naturalistic time perception task). Particularly in view of the increasing heterogeneity observed in the autistic population (Lombardo et al. 2019) and the associated lowering of the threshold for a diagnosis (Mottron and Bzdok 2020), the development of such adaptive and ecologically valid tasks should be a priority in future research. Moreover, there is a need to develop testing tools for non‐speaking individuals, who were excluded from this study due to a lack of testing options. In addition, when using computer‐based tasks, the impact of prior computer experience should be considered, as not all participants with ID are accustomed to computer keys. A key area for future research could be longitudinal studies examining the development of PM performance across the lifespan in autistic individuals with and without ID to ascertain whether there is a change in PM performance over lifetime. Moreover, the practical implementation of these results could form the basis for further research, e.g., the targeted promotion of time perception to enhance PM, an ability that is highly pertinent to everyday life.

## 5 Conclusion

In conclusion, participants across the wider autistic spectrum demonstrated comparable performance in event‐ and time‐based PM when compared with age‐ and non‐verbal ability‐matched controls, after controlling for verbal abilities. Time perception of autistic adults was lower, compared to neurotypical controls, in the naturalistic, but not in the comprehensive time perception task. A better performance in time reproduction was associated with better event‐ and time‐based PM performance in both groups. The present findings provide new insights into PM performance and time perception of autistic adults and may offer a foundation for practical interventions (e.g., clock training to improve daily planning) to offer targeted assistance to autistic individuals in their daily lives. This is one of the first studies investigating PM in autistic adults, including those with ID. The successful inclusion of participants with ID demonstrates that cognitive research can be effectively adapted for this under‐represented group. These findings provide a necessary baseline for understanding the full heterogeneity of cognitive capabilities in autism.

## Funding

This work was supported by “Stiftung Irene”.

## Conflicts of Interest

The authors declare no conflicts of interest.

## Data Availability

The data that support the findings of this study are available on request from the corresponding author. The data are not publicly available due to privacy or ethical restrictions.

## References

[aur70250-bib-0001] Altgassen, M. , M. Kliegel , P. Rendell , J. D. Henry , and J. Zöllig . 2008. “Prospective Memory in Schizophrenia: The Impact of Varying Retrospective‐Memory Load.” Journal of Clinical and Experimental Neuropsychology 30, no. 7: 777–788. 10.1080/13803390701779552. 18608664

[aur70250-bib-0002] Altgassen, M. , N. Koban , and M. Kliegel . 2012. “Do Adults With Autism Spectrum Disorders Compensate in Naturalistic Prospective Memory Tasks?” Journal of Autism and Developmental Disorders 42, no. 10: 2141–2151. 10.1007/s10803-012-1466-3. 22350339

[aur70250-bib-0004] American Psychiatric Association . 2013. Diagnostic and Statistical Manual of Mental Disorders. American Psychiatric Association. 10.1176/appi.books.9780890425596.

[aur70250-bib-0005] Block, R. A. , and S. Grondin . 2014. “Timing and Time Perception: A Selective Review and Commentary on Recent Reviews.” Frontiers in Psychology 5: 648. 10.3389/fpsyg.2014.00648. 25120497 PMC4114294

[aur70250-bib-0006] Bölte, S. , F. Poustka , and M. Rutter . 2006. Fragebogen zur sozialen Kommunikation: Autismus Screening; FSK. Huber.

[aur70250-bib-0007] Brandimonte, M. A. , G. O. Einstein , and M. A. McDaniel . 1996. Prospective Memory: Theory and Applications. Psychology Press. 10.4324/9781315806488.

[aur70250-bib-0008] Brenner, L. A. , V. H. Shih , N. L. Colich , C. A. Sugar , C. E. Bearden , and M. Dapretto . 2015. “Time Reproduction Performance Is Associated With Age and Working Memory in High‐Functioning Youth With Autism Spectrum Disorder.” Autism Research 8, no. 1: 29–37. 10.1002/aur.1401. 25078724 PMC4312276

[aur70250-bib-0009] Brodeur, D. A. , C. Gordon Green , H. Flores , and J. A. Burack . 2014. “Time Estimation Among Low‐Functioning Individuals With Autism Spectrum Disorders: Evidence of Poor Sensitivity to Variability of Short Durations.” Autism Research 7, no. 2: 237–244. 10.1002/aur.1364. 24574256

[aur70250-bib-0010] Casassus, M. , E. Poliakoff , E. Gowen , D. Poole , and L. A. Jones . 2019. “Time Perception and Autistic Spectrum Condition: A Systematic Review.” Autism Research 12, no. 10: 1440–1462. 10.1002/aur.2170. 31336032 PMC6852160

[aur70250-bib-0011] Chakrabarti, B. 2017. “Commentary: Critical Considerations for Studying Low‐Functioning Autism.” Journal of Child Psychology and Psychiatry 58, no. 4: 436–438. 10.1111/jcpp.12720. 28346760

[aur70250-bib-0012] Charlton, R. A. , G. A. McQuaid , N. R. Lee , and G. L. Wallace . 2023. “Self‐Reported Prospective and Retrospective Memory Among Middle Aged and Older Autistic and Non‐Autistic People.” Journal of Autism and Developmental Disorders 55: 1988–1994. 10.1007/s10803-023-06131-2. 37751094 PMC12076292

[aur70250-bib-0013] Cohen, S. , R. Conduit , S. W. Lockley , S. M. Rajaratnam , and K. M. Cornish . 2014. “The Relationship Between Sleep and Behavior in Autism Spectrum Disorder (ASD): A Review.” Journal of Neurodevelopmental Disorders 6, no. 1: 44. 10.1186/1866-1955-6-44. 25530819 PMC4271434

[aur70250-bib-0014] Dehnavi, F. , and A. Khan . 2024. “Time‐Based and Event‐Based Prospective Memory in Adults With Autism Spectrum Disorder: A Virtual Week Investigation.” Journal of Autism and Developmental Disorders 54, no. 6: 2298–2306. 10.1007/s10803-023-05975-y. 37079178

[aur70250-bib-0015] Desaunay, P. , A. R. Briant , D. M. Bowler , et al. 2020. “Memory in Autism Spectrum Disorder: A Meta‐Analysis of Experimental Studies.” Psychological Bulletin 146, no. 5: 377–410. 10.1037/bul0000225. 32191044

[aur70250-bib-0016] Einstein, G. O. , and M. A. McDaniel . 1996. “Retrieval Processes in Prospective Memory: Theoretical Approaches and Some New Empirical Findings.” In Prospective Memory: Theory and Applications, 115–141. Lawrence Erlbaum Associates Publishers.

[aur70250-bib-0017] Ellis, J. 1996. “Prospective Memory or the Realization of Delayed Intentions: A Conceptual Framework for Research.” In Prospective Memory: Theory and Applications, 1–22. Lawrence Erlbaum Associates.

[aur70250-bib-0018] Falter, C. M. , V. Noreika , J. H. Wearden , and A. J. Bailey . 2012. “More Consistent, Yet Less Sensitive: Interval Timing in Autism Spectrum Disorders.” Quarterly Journal of Experimental Psychology 65, no. 11: 2093–2107. 10.1080/17470218.2012.690770. 22800511

[aur70250-bib-0019] Faustmann, L. L. , and M. Altgassen . 2024. “Practice Is the Best of All Instructors‐Effects of Enactment Encoding and Episodic Future Thinking on Prospective Memory Performance in High‐Functioning Adults With Autism Spectrum Disorder.” Autism Research: Official Journal of the International Society for Autism Research 17, no. 6: 1258–1275. 10.1002/aur.3165. 38800974

[aur70250-bib-0020] Faustmann, L. L. , and M. Altgassen . 2025. “Prospective Memory Performance of Autistic Adults in Everyday Life: The Role of Stress and Motivation.” Autism Research 18, no. 7: 1447–1460. 10.1002/aur.70057. 40439024 PMC12279003

[aur70250-bib-0021] Glicksohn, J. , and M. S. Myslobodsky . 2006. Timing the Future: The Case for A Time‐Based Prospective Memory. World Scientific.

[aur70250-bib-0022] Groenman, A. P. , C. Torenvliet , T. A. Radhoe , et al. 2024. “Remembering the Future; Prospective Memory Across the Autistic Adult's Life Span.” Autism 28, no. 9: 2254–2266. 10.1177/13623613231225489. 38240223 PMC11403918

[aur70250-bib-0023] Hull, L. , K. V. Petrides , and W. Mandy . 2020. “The Female Autism Phenotype and Camouflaging: A Narrative Review.” Review Journal of Autism and Developmental Disorders 7, no. 4: 306–317. 10.1007/s40489-020-00197-9.

[aur70250-bib-0024] Jack, A. , and K. A. Pelphrey . 2017. “Annual Research Review: Understudied Populations Within the Autism Spectrum – Current Trends and Future Directions in Neuroimaging Research.” Journal of Child Psychology and Psychiatry 58, no. 4: 411–435. 10.1111/jcpp.12687. 28102566 PMC5367938

[aur70250-bib-0025] Jurek, L. , Y. Longuet , M. Baltazar , et al. 2019. “How Did I Get So Late So Soon? A Review of Time Processing and Management in Autism.” Behavioural Brain Research 374: 112121. 10.1016/j.bbr.2019.112121. 31376445

[aur70250-bib-0026] Kamp‐Becker, I. , S. Stroth , and T. Stehr . 2020. “Autismus‐Spektrum‐Störungen im Kindes‐ und Erwachsenenalter: Diagnose und Differenzialdiagnosen.” Der Nervenarzt 91, no. 5: 457–470. 10.1007/s00115-020-00901-4. 32303788

[aur70250-bib-0027] Khan, A. , N. K. Sharma , and S. Dixit . 2008. “Cognitive Load and Task Condition in Event‐ and Time‐Based Prospective Memory: An Experimental Investigation.” Journal of Psychology 142, no. 5: 517–532. 10.3200/JRLP.142.5.517-532. 18959223

[aur70250-bib-0028] Kliegel, M. , and T. Jäger . 2006. “Die Entwicklung des prospektiven Gedächtnisses über die Lebensspanne.” Zeitschrift für Entwicklungspsychologie Und Pädagogische Psychologie 38, no. 4: 162–174. 10.1026/0049-8637.38.4.162.

[aur70250-bib-0029] Kliegel, M. , M. Martin , M. A. McDaniel , and G. O. Einstein . 2002. “Complex Prospective Memory and Executive Control of Working Memory: A Process Model.” Psychologische Beiträge 44, no. 2: 303.

[aur70250-bib-0030] Kretschmer, A. , S.‐A. Lampmann , and M. Altgassen . 2014. “Relations Between Moral Reasoning, Theory of Mind and Executive Functions in Children With Autism Spectrum Disorders.” International Journal of Developmental Disabilities 60, no. 3: 174–183. 10.1179/2047387714Y.0000000045.

[aur70250-bib-0031] Landsiedel, J. , and D. M. Williams . 2020. “Increasing Extrinsic Motivation Improves Time‐Based Prospective Memory in Adults With Autism: Relations With Executive Functioning and Mentalizing.” Journal of Autism and Developmental Disorders 50, no. 4: 1133–1146. 10.1007/s10803-019-04340-2. 31865493 PMC7101298

[aur70250-bib-0032] Landsiedel, J. , D. M. Williams , and K. Abbot‐Smith . 2017. “A Meta‐Analysis and Critical Review of Prospective Memory in Autism Spectrum Disorder.” Journal of Autism and Developmental Disorders 47, no. 3: 646–666. 10.1007/s10803-016-2987-y. 28116668 PMC5352792

[aur70250-bib-0033] Lombardo, M. V. , M.‐C. Lai , and S. Baron‐Cohen . 2019. “Big Data Approaches to Decomposing Heterogeneity Across the Autism Spectrum.” Molecular Psychiatry 24, no. 10: 1435–1450. 10.1038/s41380-018-0321-0. 30617272 PMC6754748

[aur70250-bib-0034] Mackinlay, R. J. , M. Kliegel , and T. Mäntylä . 2009. “Predictors of Time‐Based Prospective Memory in Children.” Journal of Experimental Child Psychology 102, no. 3: 251–264. 10.1016/j.jecp.2008.08.006. 19091327

[aur70250-bib-0035] Maister, L. , and K. C. Plaisted‐Grant . 2011. “Time Perception and Its Relationship to Memory in Autism Spectrum Conditions.” Developmental Science 14, no. 6: 1311–1322. 10.1111/j.1467-7687.2011.01077.x. 22010891

[aur70250-bib-0036] Matthews, W. J. , and W. H. Meck . 2016. “Temporal Cognition: Connecting Subjective Time to Perception, Attention, and Memory.” Psychological Bulletin 142, no. 8: 865–907. 10.1037/bul0000045. 27196725

[aur70250-bib-0037] McBride, D. M. , and M. Flaherty . 2020. “Comparing Costs in Time‐Based and Event‐Based Prospective Memory.” Memory 28, no. 7: 918–925. 10.1080/09658211.2020.1798463. 32701016

[aur70250-bib-0038] McDaniels, M. A. , and G. O. Einstein . 2000. “Strategic and Automatic Processes in Prospective Memory Retrieval: A Multiprocess Framework.” Applied Cognitive Psychology 14, no. SpecIssue: S127–S144. 10.1002/acp.775.

[aur70250-bib-0039] Mioni, G. , S. Santon , F. Stablum , and C. Cornoldi . 2017. “Time‐Based Prospective Memory Difficulties in Children With ADHD and the Role of Time Perception and Working Memory.” Child Neuropsychology: A Journal on Normal and Abnormal Development in Childhood and Adolescence 23, no. 5: 588–608. 10.1080/09297049.2016.1172561. 27094171

[aur70250-bib-0040] Mioni, G. , and F. Stablum . 2014. “Monitoring Behaviour in a Time‐Based Prospective Memory Task: The Involvement of Executive Functions and Time Perception.” Memory 22, no. 5: 536–552. 10.1080/09658211.2013.801987. 23734633

[aur70250-bib-0041] Mioni, G. , F. Stablum , S. M. McClintock , and A. Cantagallo . 2012. “Time‐Based Prospective Memory in Severe Traumatic Brain Injury Patients: The Involvement of Executive Functions and Time Perception.” Journal of the International Neuropsychological Society: JINS 18, no. 4: 697–705. 10.1017/S1355617712000306. 22433779

[aur70250-bib-0042] Mottron, L. , and D. Bzdok . 2020. “Autism Spectrum Heterogeneity: Fact or Artifact?” Molecular Psychiatry 25, no. 12: 3178–3185. 10.1038/s41380-020-0748-y. 32355335 PMC7714694

[aur70250-bib-0043] Poole, D. , M. Casassus , E. Gowen , E. Poliakoff , and L. A. Jones . 2022. “Time Perception in Autistic Adults: Interval and Event Timing Judgments Do Not Differ From Nonautistics.” Journal of Experimental Psychology: General 151, no. 11: 2666–2682. 10.1037/xge0001203. 35467931

[aur70250-bib-0044] Poole, D. , E. Gowen , E. Poliakoff , and L. A. Jones . 2021. “‘No Idea of Time’: Parents Report Differences in Autistic Children's Behaviour Relating to Time in a Mixed‐Methods Study.” Autism 25, no. 6: 1797–1808. 10.1177/13623613211010014. 33926273 PMC8323338

[aur70250-bib-0045] Ring, M. , S. B. Gaigg , and D. M. Bowler . 2016. “Relational Memory Processes in Adults With Autism Spectrum Disorder.” Autism Research 9, no. 1: 97–106. 10.1002/aur.1493. 25952759

[aur70250-bib-0046] Rutter, M. , A. Bailey , and C. Lord . 2003. Scq. The Social Communication Questionnaire, 5. Western Psychological Services. https://www.renartlivros.com.br/conteudos.renartlivros.com.br/2P5900/Extracto%20del%20manual%20SCQ.pdf.

[aur70250-bib-0047] Sheppard, D. P. , J. P. Bruineberg , A. Kretschmer‐Trendowicz , and M. Altgassen . 2018. “Prospective Memory in Autism: Theory and Literature Review.” Clinical Neuropsychologist 32, no. 5: 748–782. 10.1080/13854046.2018.1435823. 29536800

[aur70250-bib-0048] Sheppard, D. P. , L. Kvavilashvili , and N. Ryder . 2016. “Event‐Based Prospective Memory in Mildly and Severely Autistic Children.” Research in Developmental Disabilities 49–50: 22–33. 10.1016/j.ridd.2015.09.018. 26647004

[aur70250-bib-0049] Shi, Z. , S. Ganzenmüller , and H. J. Müller . 2013. “Reducing Bias in Auditory Duration Reproduction by Integrating the Reproduced Signal.” PLoS One 8, no. 4: e62065. 10.1371/journal.pone.0062065. 23614014 PMC3628943

[aur70250-bib-0050] Üstün, S. , E. H. Kale , and M. Çiçek . 2017. “Neural Networks for Time Perception and Working Memory.” Frontiers in Human Neuroscience 11: 83. 10.3389/fnhum.2017.00083. 28286475 PMC5324352

[aur70250-bib-0051] Waizbard‐Bartov, E. , D. Fein , C. Lord , and D. G. Amaral . 2023. “Autism Severity and Its Relationship to Disability.” Autism Research 16, no. 4: 685–696. 10.1002/aur.2898. 36786314 PMC10500663

[aur70250-bib-0052] Wallace, G. L. , and F. Happé . 2008. “Time Perception in Autism Spectrum Disorders.” Research in Autism Spectrum Disorders 2, no. 3: 447–455. 10.1016/j.rasd.2007.09.005.

[aur70250-bib-0053] Wechsler, D. 2008. “Wechsler Adult Intelligence Scale—Forth Edition.” Archives of Clinical Neuropsychology. 10.1037/t15169-000.

[aur70250-bib-0055] Williams, D. M. , C. Jarrold , C. Grainger , and S. E. Lind . 2014. “Diminished Time‐Based, but Undiminished Event‐Based, Prospective Memory Among Intellectually High‐Functioning Adults With Autism Spectrum Disorder: Relation to Working Memory Ability.” Neuropsychology 28, no. 1: 30–42. 10.1037/neu0000008. 24128041 PMC3906801

[aur70250-bib-0056] Wójcik, M. , J. Neckar , and A. Niedźwieńska . 2022. “Predictors of Everyday Prospective Memory Performance: A Superiority in the Execution of Event‐Based Tasks Over Time‐Based Tasks Reverses in Real‐Life Situations.” Journal of Applied Research in Memory and Cognition 11, no. 2: 245–257. 10.1037/h0101872.

[aur70250-bib-0057] Zeidan, J. , E. Fombonne , J. Scorah , et al. 2022. “Global Prevalence of Autism: A Systematic Review Update.” Autism Research : Official Journal of the International Society for Autism Research 15, no. 5: 778–790. 10.1002/aur.2696. 35238171 PMC9310578

